# Singular Spectrum Analysis for Modal Estimation from Stationary Response Only

**DOI:** 10.3390/s22072585

**Published:** 2022-03-28

**Authors:** Chang-Sheng Lin, Yi-Xiu Wu

**Affiliations:** Department of Vehicle Engineering, National Pingtung University of Science and Technology, Pingtung 912301, Taiwan; m10938002@mail.npust.edu.tw

**Keywords:** singular spectrum analysis, state space, Hankel matrix, modal estimation

## Abstract

Conventional experimental modal analysis uses excitation and response information to estimate the frequency response function. However, many engineering structures face excitation signals that are difficult to measure, so output-only modal estimation is an important issue. In this paper, singular spectrum analysis is employed to construct a Hankel matrix of appropriate dimensions based on the measured response data, and the observability of the system state space model is used to treat the Hankel matrix as three components containing system characteristics, excitation and noise. Singular value decomposition is used to factorize the data matrix and use the characteristics of the left and right singular matrices to reduce the dimension of the data matrix to improve calculation efficiency. Furthermore, the singular spectrum is employed to estimate the minimum order to reconstruct the Hankel matrix; then, the excitation and noise components can be removed, and the system observability matrix can be obtained. By appropriately a factorizing system observability matrix, we obtain the system matrix to estimate the modal parameters. In addition, the fictitious modes produced by increasing the order of the matrix can be eliminated through the stabilization diagram.

## 1. Introduction

In the design of civil structures or mechanical systems, the actual working conditions such as earthquakes on buildings, the rotational speed of shafts and the operation of machine tools are often considered. If the Fourier spectrum of operational conditions has rich frequency content around the structure modes, it will cause the structure to resonate and reduce the life cycle of the structure, so the industry will include modal analysis as a reference in the design. The traditional experimental modal analysis (EMA) belongs to the frequency-domain analysis method. The dynamic information of systems extracted from frequency response function can be obtained by knowing both the excitation and response of a system. Meanwhile, in structural systems with small damping and without severe modal interference, the identification results are good in general. However, in many practical engineering structures, the structure often vibrates under unknown ambient excitation, which is often difficult to measure. Therefore, how to estimate the modal parameters of such structural systems subjected to unknown excitation for further structural control, damage detection or finite element model updating is a very important issue in structural engineering.

In 1965, Ho and Kalman [1] proposed the minimum realization algorithm, which uses Markov parameters composed of impulse response functions to obtain the state space representation of the minimum order. This method can accurately describe the system; it does not, however, effectively work on assessment with noise influence. In 1974, Zeiger and McEwen [2] proposed the concept of combining singular value decomposition (SVD) with the minimum realization method; in the 1980s, Juang and Pappa [3] developed the Eigensystem Realization Algorithm (ERA) on the basis of SVD and the minimum realization method. The method realizes the system with the minimum order and applies it to modal estimation. Later, Juang et al. further improved ERA and proposed the so-called Eigensystem Realization Algorithm using Data Correlation (ERA/DC) [4]. This method uses data correlation to reduce noise under the theoretical framework of ERA, thereby improving the accuracy of modal identification. In addition, Principal Component Analysis (PCA) is used to solve system identification problems where the number of degrees of freedom (DOF) of a system is less than the number of measurement response channel. Since in the modal coordinates, the random vibration response of the low-damping structure can be decomposed into the inner product of the modal shape and the modal response, and the natural frequency and damping ratio can be obtained by the single DOF modal identification method. Therefore, for operating modal analysis (OMA) based on principal component analysis (PCA), it examines the relationship between the transformation matrix and the mode shape matrix, estimates the correlation between the principal component matrix and the modal response matrix, and transforms the Operational Modal Analysis (OMA) problem into a PCA for the stationary response data of low-damped structures. In 1986, Leuridan et al. [5] used a multivariate model in the form of inhomogeneous finite difference equations to identify the modal parameters of mechanical structures. By applying the multiple-input multiple-output (MIMO) concept, the modal parameters of the system can be completely estimated from the response data, including vibration signals with highly-coupled and repeated modes. Guan et al. [6] systematically compared four algorithms such as PCA, ICA, SOBI and LLE and analyzed their effectiveness in modal estimation. To improve the computational efficiency, Zhang et al. [7] proposed a simplified data analysis process based on PCA to estimate the order of the system to be identified and the corresponding range of frequencies contained in the signal, so as to avoid performing wavelet transform (WT) in the lower-contribution frequency bandwidth. Moreover, they proposed a modal estimation algorithm based on continuous wavelet transform (CWT). Yang and Nagarajaiah [8] used Sparse Component Analysis (SCA) to decompose the signal using the sparseness in the time-frequency transform domain and discussed the effectiveness of modal identification using measurement signals with insufficient modal information. The essence of modal expansion can show that the response of the sparser modal frequency domain information will naturally gather into the column vector of the modal matrix, and then estimate the modal information; this method has been effectively applicable to the parametric identification of actual bridge structures [9].

Singular Spectrum Analysis (SSA) [10] is a signal decomposition technique, through four steps, Embedding, Singular Value Decomposition (SVD), Grouping and Averaging, used to find the trend function of the signal, remove the influence of noise to smooth the signal and decompose the original signal into components with trend and periodicity. This method combines time series analysis, multivariate statistics and geometry, dynamic systems and signal processing and is widely used in statistics, multivariate geometric analysis, dynamic systems and signal processing. SSA is a commonly used non-parametric solution method in time series and multivariate statistical analysis [11] and has been widely used in marine and meteorological forecasting and diagnosis [12]. Liu et al. [13] combined the sensitivity-based damage identification method with SSA analysis to improve the damage identification results. Luo et al. [14] used SSA for noise reduction in vibration signals and combined other methods to judge rotor faults. Lakshmi et al. [15] used the ARMAX model to analyze structural damage; due to environmental or noise factors, the signal characteristics of damage would be inconspicuous, so SSA was used to reconstruct the signal to remove the noise and enhance the ARMAX recognition results. Li et al. [16] used Blind Signal Separation (BSS) to analyze the modal parameters of bridge structures, and introduced SSA to filter out the results of non-main features, thereby overcoming the difficulty of covering up the main features of the structure due to vehicles traveling on the bridge. Al-Bugharbee et al. [17] used SSA to preprocess the signal to reduce noise, and then analyze the rotor faults. Prawin et al. [18] studied the effect of Breathing cracks on cantilever beams and used SSA combined with a damage index to locate the possible damage in a structure. Li et al. [19] conducted a spectrum analysis on the measured responses of arch dams excited by earthquakes and used ensemble empirical mode decomposition combined with a wavelet threshold and a singular spectrum analysis to filter and highlight the dynamic characteristics of arch dams. Fitzgerald et al. [20] designed a piezoelectric energy-harvesting device (EHD) in the form of a cantilever beam and used the SSA method to filter relevant interference signals, thereby accurately obtaining the signal of the measured object. Trendafilova [21] performed SSA on the impulse response of a single degree of freedom in the beam structure, decomposed the impulse response into linear and nonlinear oscillatory signals by using different orders of a singular spectrum and selected a proper order of singular spectrum to reconstruct the original signal. In general, SSA is equivalent to low-pass filtering the original signal, filtering out high-frequency noise and aperiodic signals in the signal and then smoothing the original signal so as to extract the information that mainly contributes to the dynamic characteristics of the system. This method means that SSA can preserve the characteristics of important dynamic parameters of the system in the signal and remove the irregular and random components in the time series.

This research mainly uses singular spectrum analysis (SSA) with state space for output-only system modal parameter estimation. We construct the original response data into a Hankel matrix, for which the singular spectrum to estimate the main singular values of the matrix, and use the grouping and averaging in the SSA procedure, respectively, to remove the singular values with lower contributions and reconstruct a Hankel matrix. By introducing the observability property of the state space, a Hankel matrix with appropriate dimensions is applicable to reduce the influence of excitation and noise on the response by singular spectrum analysis, thereby effectively improving the accuracy of system identification. Finally, numerical simulations confirm the validity and reliability of singular spectrum analysis for modal estimation.

## 2. Research Methods

Singular Spectrum Analysis (SSA) is a signal decomposition method. This method is used to decompose the original signal into multiple components, determine the components to find the signal trend, eliminate the influence of noise, reconstruct the signal to make it smooth, and can determine the order of the system. The four steps singular spectrum analysis are embedding, singular value decomposition (SVD), grouping and averaging.

### 2.1. State Space Equation

A state space equation is applicable to describe a physical system in terms of inputs, outputs and states. This paper uses a linear time-invariant system and starts by considering the measurement data contaminated with noise in practical engineering. The discrete state space equation can be expressed as:

(1) $${\underline{x}}_{k+1}=\;\left[A\right]{\underline{x}}_{k}+\;\left[B\right]{\underline{u}}_{k}+{\underline{w}}_{k}$$

(2) $${\underline{y}}_{k}=\;\left[C\right]{\underline{x}}_{k}+{\underline{v}}_{k}=\;\left[C\right]{\left[A\right]}^{k}{\underline{x}}_{0}+\overset{k-1}{\underset{i=0}{\sum }}\left[C\right]{\left[A\right]}^{k-i-1}\left[B\right]{\underline{u}}_{i}+{\underline{v}}_{k}$$

Equation (1) is the state space equation, where the system motion equation is described by the state vector ${\underline{x}}_{k}$, state matrix $\left[A\right]$ and input matrix $\left[B\right]$. Equation (2) is the influence of the system state on the actual output; ${\underline{x}}_{k}$ and ${\underline{y}}_{k}$ are the state and output vectors at time instant k, respectively, ${\underline{u}}_{k}$ is the input vector and ${\underline{w}}_{k}$ and ${\underline{v}}_{k}$ are noise vectors. $\left[A\right]$, $\left[B\right]$ and $\left[C\right]$ are the coefficient matrices of the motion equation in the form of state variables, where $\left[A\right]$ is the system matrix containing the dynamic information of the system. One property in the state space is observability, which represents the state matrix of the system inferring from the output data. The output equation based on observability can be expressed as:

(3) $$\left[\begin{array}{c} {\underline{y}}_{0}\\ {\underline{y}}_{1}\\ \vdots \\ {\underline{y}}_{k} \end{array}\right]=\left[\begin{array}{c} \left[C\right]{\underline{x}}_{0}\\ \left[C\right]{\underline{x}}_{1}\\ \vdots \\ \left[C\right]{\underline{x}}_{k} \end{array}\right]=\left[\begin{array}{c} \left[C\right]{\underline{x}}_{0}\\ \left[C\right]\left[A\right]{\underline{x}}_{0}\\ \vdots \\ \left[C\right]{\left[A\right]}^{k}{\underline{x}}_{0} \end{array}\right]=\;\left[\alpha \right]{\underline{x}}_{0}$$

where $\left[\alpha \right]$ is called the observability matrix, which represents the matrix of the initial state of the system at each moment of the system. By factorizing the observability matrix into different time series, the system matrix $\left[A\right]$ can then be obtained.

### 2.2. Singular Spectrum Analysis

**Embedding**

Embedding is to create a trajectory matrix from the original signal. The trajectory matrix is used to arrange the measurement response signals in sections. The anti-diagonal elements correspond to the same time point, and the representative trajectory matrix is the Hankel matrix.

Consider a known time signal with measurement degrees of freedom p and signal length m, and the data matrix can be represented as $Y_{p\times m}=\left[\begin{array}{cccc} {\underline{y}}_{1} & {\underline{y}}_{2} & \cdots & {\underline{y}}_{m} \end{array}\right]$. We used this signal to perform segmental arrangement to create a Hankel matrix H, where the number of row vectors is K and the number of column vectors is L, as follows:

(4) $$\begin{array}{c} H={\left[\begin{array}{cccc} {\underline{y}}_{1} & {\underline{y}}_{2} & \cdots & {\underline{y}}_{K}\\ {\underline{y}}_{2} & {\underline{y}}_{3} & \cdots & {\underline{y}}_{K+1}\\ \vdots & \vdots & \ddots & \vdots \\ {\underline{y}}_{L} & {\underline{y}}_{L+1} & \cdots & {\underline{y}}_{L+K-1} \end{array}\right]}_{pL\times K}\\ \;\;\;\;=\left[\begin{array}{c} C\\ CA\\ \vdots \\ CA^{L} \end{array}\right]\left[\begin{array}{cccc} {\underline{x}}_{1} & {\underline{x}}_{2} & \cdots & {\underline{x}}_{L+K-1} \end{array}\right]+\left[\begin{array}{cccc} CB & 0 & \cdots & 0\\ CAB & CB & 0 & \vdots \\ \vdots & \vdots & \ddots & 0\\ CA^{L}B & CA^{L-1}B & \cdots & CB \end{array}\right]\left[\begin{array}{cccc} {\underline{u}}_{1} & {\underline{u}}_{2} & \cdots & {\underline{u}}_{K}\\ {\underline{u}}_{2} & {\underline{u}}_{3} & \cdots & {\underline{u}}_{K+1}\\ \vdots & \vdots & \ddots & \vdots \\ {\underline{u}}_{L} & {\underline{u}}_{L+1} & \cdots & {\underline{u}}_{L+K-1} \end{array}\right]+\left[\bar{v}\right]\\ \;\;\;\;=\left[\alpha \right]\left[{\hat{X}}_{k}\right]+\left[L\right]\left[U\right]+\left[\bar{v}\right] \end{array}$$

where $\left[\alpha \right]$ and $\left[{\hat{X}}_{k}\right]$ are the observability and state matrices of the system, respectively, and $\left[U\right]$ is the Hankel matrix of the external force. If the randomness of noise and excitation is included in the response data, it is impossible to obtain accurate dynamic characteristics by using the response data only. Therefore, the influence of noise needs to be eliminated first. The noise may include measurement noise or model errors. Since the noise has no correlation with the dynamic characteristics of the system, the predecessors often assumed that the noise is stationary white, so that the noise term $\left[\bar{v}\right]$ is uncorrelated to the dynamic characteristics of a system. To avoid the column vector not being extended enough to the original data length, the dimension of the Hankel matrix would be constructed under the restrictions of $L\leq \frac{m}{2}$ and L≤K. Equation (4) indicates that the constructed Hankel matrix is composed of the information of structure itself, excitation and noise.

**Singular Value Decomposition**

Singular value decomposition (SVD) is a common decomposition of non-square matrix, which can decompose a matrix into multiple combinations, and is often applicable to signal processing and statistics fields. Through SVD, the factorization of H can be expressed as:

(5) $$\begin{array}{cc} H_{pL\times k} & =U_{pL\times pL}\Sigma_{pL\times K}V^{T}{}_{K\times K}\\ & =\left[\begin{array}{ccc} {\underline{u}}_{1} & \cdots & {\underline{u}}_{pL} \end{array}\right]\left[\begin{array}{cccc} s_{1} & 0 & 0 & 0\\ 0 & \ddots & 0 & 0\\ 0 & 0 & s_{pL} & 0 \end{array}\right]\left[\begin{array}{c} {\underline{v}}_{1}^{T}\\ \vdots \\ {\underline{v}}_{K}^{T} \end{array}\right] \end{array}$$

where U and V are orthogonal matrices, and Σ is a singular value matrix consisting of zero off-diagonal entries and obvious non-zero singular values on the main diagonal entries. By arranging the singular values from large to small, i.e., $s_{1}{>s}_{2}>\cdots s_{pL}$, to obtain the singular spectrum of the signal, the relatively large singular value can be determined as the system component; otherwise, the smaller singular value will be viewed as noise.

To measure a more complete system signal, a longer acquisition time sample needs to be chosen, so the corresponding operation time due to singular value decomposition is increased. In general, the length K of the reference signal will be much larger than matrix column pL. To reduce the operation time of singular value decomposition, we construct the data matrix $H_{pL\times K}H_{pL\times K}^{T}$ in conjunction with the characteristics of the left and right singular matrices, and the dimension of the $H_{pL\times K}H_{pL\times K}^{T}$ is reduced from pL×K to pL×pL as follows:

(6) $$\begin{array}{cc} H_{pL\times K}H_{pL\times K}^{T} & =U_{pL\times pL}\Sigma_{pL\times K}V^{T}{}_{K\times K}V_{K\times K}\Sigma_{pL\times K}^{T}U^{T}{}_{pL\times pL}\\ & =U_{pL\times pL}\Sigma_{pL\times pL}^{2}U^{T}{}_{pL\times pL} \end{array}$$

Subsequently, the right orthogonal matrix $V_{K\times K}^{T}$ corresponding to the matrix $H_{pL\times k}$ is evaluated as follows:

(7) $$V_{K\times K}^{T}={\left[\begin{array}{c} \Sigma_{pL\times pL}^{-1}\\ 0_{(K-pL)\times pL} \end{array}\right]}_{K\times pL}U^{T}{}_{pL\times pL}H_{pL\times K}$$

Through the matrix dimension reduction, we can define the Hankel matrix parameter K as m−L+1, so that the parameters required to be adjusted are reduced in the later analysis, and then the system order is estimated by the stabilization diagram.

**Grouping**

By comparing the corresponding values in the singular spectrum, the order corresponding to the line segment with a larger slope in the singular spectrum is evaluated as the selected system order. The vectors of the corresponding order are employed to reconstruct the Hankel matrix, and then the singular value decomposition is performed as follows:

(8) $$H_{pL\times K}=\;\left(\begin{array}{cc} \left[U_{1}\right] & \left[U_{2}\right] \end{array}\right)\left(\begin{array}{cc} \left[S_{1}\right] & 0\\ 0 & \left[S_{2}\right] \end{array}\right)\left(\begin{array}{c} {\left[V_{1}\right]}^{T}\\ {\left[V_{2}\right]}^{T} \end{array}\right)$$

where ${\left[S_{1}\right]}_{i\times i}$ determines the order of the matrix to be retained, and ${\left[S_{2}\right]}_{\left(pL-i\right)\times \left(pL-K\right)}$ determines the order of the matrix to be removed. The first few items with larger singular values are used to reconstruct a new Hankel matrix $\left[\hat{H}\right]$, so as to retain the dynamic characteristics of the system and reduce the excitation and noise components:

(9) $$\begin{array}{cc} \left[\hat{H}\right] & ={\left[U_{1}\right]}_{pL\times i}{\left[S_{1}\right]}_{i\times i}{\left[V_{1}\right]}_{i\times K}^{T}\\ & ={\left[\begin{array}{cccc} {\tilde{\underline{y}}}_{1} & {\tilde{\underline{y}}}_{2} & \cdots & {\tilde{\underline{y}}}_{K}\\ {\tilde{\underline{y}}}_{2} & {\tilde{\underline{y}}}_{3} & \cdots & {\tilde{\underline{y}}}_{K+1}\\ \vdots & \vdots & \ddots & \vdots \\ {\tilde{\underline{y}}}_{L} & {\tilde{\underline{y}}}_{L+1} & \cdots & {\tilde{\underline{y}}}_{L+K-1} \end{array}\right]}_{pL\times K} \end{array}$$

where $\left[\hat{H}\right]$ is the reconstructed Hank matrix, and i is the number of pre-reserved components.

**Averaging**

In the process of reconstructing the new Hank matrix $\left[\hat{H}\right]$, the calculation of SVD will result in that the responses of the anti-diagonals will not be exactly equal. Since this matrix does not conform to the form of the Hank matrix, it is necessary to average the values of the anti-diagonals. We first reconstructed the signal and then reconstructed it into a Hank matrix. The correction equation is as follows:

(10) $${\underline{g}}_{\left(n\right)}=\;\left\{\begin{array}{c} \frac{\sum_{m=1}^{n}{\tilde{\underline{y}}}_{m,n-m+1}}{n},0\leq n<L\\ \frac{\sum_{m=1}^{L}{\underline{\tilde{y}}}_{m,n-m+1}}{L},L\leq n<K+1\\ \frac{\sum_{m=n-K+1}^{N-K+1}{\tilde{\underline{y}}}_{m,n-m+1}}{N-n+1},K+1\leq n<L+K \end{array}\right.$$

We reconstructed the above corrected time series ${\underline{g}}_{\left(n\right)}$ into a Hankel matrix, as shown in the following:

(11) $$\left[\Omega \right]\;=\;{\left[\begin{array}{cccc} {\underline{g}}_{1} & {\underline{g}}_{2} & \cdots & {\underline{g}}_{K}\\ {\underline{g}}_{2} & {\underline{g}}_{3} & \cdots & {\underline{g}}_{K+1}\\ \vdots & \vdots & \ddots & \vdots \\ {\underline{g}}_{L} & {\underline{g}}_{L+1} & \cdots & {\underline{g}}_{K+L-1} \end{array}\right]}_{pL\times K}$$

With the minimum order of the singular spectrum estimation matrix, the reconstructed Hank matrix $\left[\Omega \right]$ has eliminated the low-contribution components in the signal and then eliminated most of the excitation and noise information in the signal. By comparing Equation (11) with Equation (4) and removing the terms of excitation and noise, the following equation is obtained:

(12) $$\left[\Omega \right]\;=\;\left[\alpha \right]\left[{\hat{X}}_{k}\right]$$

By using the SVD, $\left[\Omega \right]$ can be factorized into $\left[\alpha \right]$ and $\left[{\hat{X}}_{k}\right]$ as follows:

(13) $$\left[\Omega \right]\;=U\Sigma V^{T}=\left[\alpha \right]\left[{\hat{X}}_{k}\right]$$

where $\left[\alpha \right]\;=U\Sigma^{1}$ and $\left[{\hat{X}}_{k}\right]\;=\Sigma^{0}V^{T}$. According to Equation (4), $\left[\alpha \right]$ contains system matrix $\left[A\right]$, which can be expressed as:

(14) $$\left[\alpha \right]\;=\left[\begin{array}{c} C\\ CA\\ \vdots \\ CA^{L-1} \end{array}\right]$$

To obtain the system matrix $\left[A\right]$, we factorize the matrix $\left[\alpha \right]$ into two different time-series matrices ${\left[\alpha \right]}_{1}$ and ${\left[\alpha \right]}_{2}$ as follows:

(15) $$\begin{array}{l} {\left[\alpha \right]}_{1}=\left[\begin{array}{c} C\\ CA\\ \vdots \\ CA^{i-1} \end{array}\right]\\ {\left[\alpha \right]}_{2}=\left[\begin{array}{c} CA\\ CA^{2}\\ \vdots \\ CA^{i} \end{array}\right] \end{array}$$

To obtain the relationship between two different time series ${\left[\alpha \right]}_{1}$ and ${\left[\alpha \right]}_{2}$, a system matrix $\left[P\right]$ is defined, which is expressed as follows:

(16) $$\left[P\right]{\left[\alpha \right]}_{1}={\left[\alpha \right]}_{2}$$

Since ${\left[\alpha \right]}_{1}$ is not a square matrix, directly using matrix inversion cannot obtain the system matrix. Therefore, the Moore–Penrose pseudoinverse method is used to perform the generalized inverse of ${\left[\alpha \right]}_{1}$, and then the inverse matrix of ${\left[\alpha \right]}_{1}$ is obtained. In practice, the singular value decomposition of ${\left[\alpha \right]}_{1}$ can be evaluated as follows:

(17) $${\left[\alpha \right]}_{1}=u\Lambda v^{T}$$

Substituting Equation (17) into Equation (16), $\left[P\right]$ can be expressed as:

(18) $$\left[P\right]\;={\left[\alpha \right]}_{2}v\Lambda^{-1}u^{T}$$

The system matrix $\left[P\right]$ obtained above contains the dynamic characteristics of the system, including natural frequencies, damping ratios and mode shapes. To obtain the dynamic characteristics, the eigenvalue decomposition of the system matrix is as follows:

(19) $$\left[P\right]\left[\Psi \right]\;=\;\left[\Psi \right]\left[Ζ\right]$$

where $\left[Ζ\right]=diag\left(\begin{array}{cccc} z_{1} & z_{2} & \cdots & z_{i} \end{array}\right)$ is the i eigenvalues of the matrix $\left[P\right]$, and $\left[\Psi \right]\;=\;\left[\begin{array}{cccc} {\underline{\psi }}_{1} & {\underline{\psi }}_{2} & \cdots & {\underline{\psi }}_{i} \end{array}\right]$ is the corresponding eigenvectors, that is, the mode shapes of the structural system. The eigenvalues of discrete system have the following relationship with the eigenvalues of the continuous vibration system:

(20) $$\lambda_{k}=\frac{1}{\Delta t}\ln z_{k}=\lambda_{k}^{R}+j\lambda_{k}^{I},\;k=1,2,\cdots ,i$$

where Δt is the resolution of the discrete signal, and $\lambda_{k}^{R}$ and $\lambda_{k}^{I}$ are the real and imaginary parts of the eigenvalue $\lambda_{k}$, respectively. Comparing with the eigenvalues of the vibration system, the natural frequency $\omega_{k}$ and damping ratio $\xi_{k}$ of the system can be obtained as follows:

(21) $$\omega_{k}=\sqrt{{\left(\lambda_{k}^{R}\right)}^{2}+{\left(\lambda_{k}^{I}\right)}^{2}}$$

(22) $$\xi_{k}=\frac{-\lambda_{k}^{R}}{\sqrt{{\left(\lambda_{k}^{R}\right)}^{2}+{\left(\lambda_{k}^{I}\right)}^{2}}}$$

### 2.3. Stabilization Diagram

In theory, a continuum structure has an infinite number of degrees of freedom and modes. In practice, we cannot accurately estimate how many modes are required to describe the observed dynamic behavior of the structural system and therefore cannot accurately estimate the system order. In recent years, with the improvement in computer performance, scholars have proposed stabilization diagrams in the field of system identification, which can effectively confirm the order of an unknown system. The method of the stabilization diagram is to use to the general structure as a second-order vibration system; if it is an underdamped system, its eigenvalues will have conjugate characteristics, which can be applicable to identify the true modes; otherwise, they are identified as fictitious modes. Meanwhile, when solving the eigenvalues of the system matrix, whether the eigenvalues evaluated under different system orders are stable or not can be applicable to identify if they belong to true modes.

By setting the error range of natural frequency $\left|\left(f_{n}-f_{n-1}\right)/f_{n}\right|<0.01$ and damping ratio $\left|\left(\xi_{n}-\xi_{n-1}\right)/\xi_{n}\right|<0.05$, where $f_{n}$ and $\xi_{n}$ are the natural frequency and damping ratio of the nth mode, respectively, we use the above error range to determine whether the n th and n+1 th modes are a conjugate pair and then use the eigenvector corresponding to each eigenvalue to further estimate the system order. The Modal Assurance Criteria (MAC) extensively used in the experimental modal analysis is employed check for agreement between the identified exact eigenvectors, i.e., mode shapes. The eigenvectors corresponding to the conjugate-pair eigenvalues are generally consistent. We use MAC to confirm whether the pole is stable, so as to assist in confirming the true structural modes. The definition of the MAC is:

(23) $$MAC(n,n+1)=\frac{{\left|{\underline{\phi }}_{n}^{H}{\underline{\phi }}_{n+1}\right|}^{2}}{\left({\underline{\phi }}_{n}^{H}{\underline{\phi }}_{n}\right)\left({\underline{\phi }}_{n+1}^{H}{\underline{\phi }}_{n+1}\right)}$$

where the superscript H is the conjugate transpose operator, and ${\underline{\phi }}_{n}$ is the *n*-th eigenvector. Furthermore, consider a structural system with complex modes, and the real and imaginary parts of the identified mode shape are denoted as ${\underline{\phi }}_{R}$ and ${\underline{\phi }}_{I}$, respectively. We evaluate the variance $S_{xx}$, $S_{yy}$ and covariance $S_{xy}$ of the eigenvectors ${\underline{\phi }}_{R}$ and ${\underline{\phi }}_{I}$ and then construct $S_{xx}$, $S_{yy}$ and $S_{xy}$ into a variance–covariance matrix S as follows:

(24) $$S=\left[\begin{array}{cc} S_{xx} & S_{xy}\\ S_{xy} & S_{yy} \end{array}\right]$$

where $S_{xx}={\underline{\phi }}_{R}^{T}{\underline{\phi }}_{R}$, $S_{yy}={\underline{\phi }}_{I}^{T}{\underline{\phi }}_{I}$ and $S_{xy}={\underline{\phi }}_{R}^{T}{\underline{\phi }}_{I}$. By solving the eigenvalue problem associated with S, the eigenvalues $\lambda_{1,2}$ of S can be obtained as follows:

(25) $$\lambda_{1,2}=\frac{S_{xx}+S_{yy}}{2}\pm \sqrt{{\left(\frac{S_{xx}-S_{yy}}{2}\right)}^{2}+S_{xy}{}^{2}}$$

The Modal Phase Collinearity (MPC) is defined by the above eigenvalues $\lambda_{1,2}$ and can be employed to identify whether the mode shape corresponds to a real mode, which is expressed as follows [22]:

(26) $$MPC({\underline{\phi }}_{n})={\left(\frac{\lambda_{1}-\lambda_{2}}{\lambda_{1}+\lambda_{2}}\right)}^{2}$$

It is known from equation (26) that the MPC values range from 0 for a mode with completely uncorrelated phase angles to 1 for a monophase result. MPC quantifies that spatial consistency of the identification results and the degree of monophase behavior by comparing the real size of the eigenvalues of S [22]. For classical normal modes, the corresponding mode shapes are real or monophase vectors, and S of the real and imaginary parts of mode shapes has only one nonzero eigenvalue; on the other hand, the $\lambda_{1}$ and $\lambda_{2}$ of S will be approximately equal if the phase angles of the identified mode shape are uncorrelated [22]. In this paper, MPC analysis is employed to identify whether the mode is a real mode. MPC is set to 0.7 as a preliminary extracting threshold to be employed to filter some fictitious modes [23], and then the real modes can be confirmed by observing the existence and changes of the poles.

## 3. Numerical Simulations and Verifications

When the structure is subjected to dynamic testing under external force excitation, the modal parameters can be estimated from the measured data of the excitation and response of the structural system. However, it is often difficult to actually perform dynamic testing of large structures, and the modal information of real structures is often difficult to measure completely. Therefore, to confirm the validity of the developed algorithm, numerical simulations are usually used to verify the feasibility of the proposed method.

### 3.1. 6-DOF Chain Model

**Figure 1:**
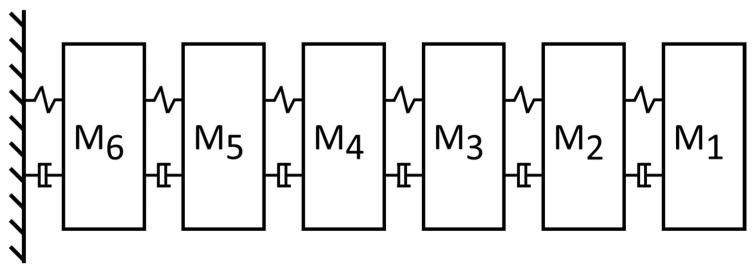
A schematic diagram of the 6-DOF chain model.

A 6-DOF linear chain model system, as shown in Figure 1, is employed to demonstrate the effectiveness of the proposed method [24], and the corresponding mass matrix $\left[M\right]$, stiffness matrix $\left[K\right]$ and damping matrix $\left[C\right]$ of the system are expressed as follows:

(27) $$\left[M\right]\;=\left[\begin{array}{cccccc} 2 & 0 & 0 & 0 & 0 & 0\\ 0 & 2 & 0 & 0 & 0 & 0\\ 0 & 0 & 2 & 0 & 0 & 0\\ 0 & 0 & 0 & 2 & 0 & 0\\ 0 & 0 & 0 & 0 & 3 & 0\\ 0 & 0 & 0 & 0 & 0 & 4 \end{array}\right]\mathrm{Kg}$$

(28) K =6001−10000−12−10000−12−10000−12−10000−13−20000−25Nm

(29) C =0.1M+0.001K N⋅sm

**Figure 2:**
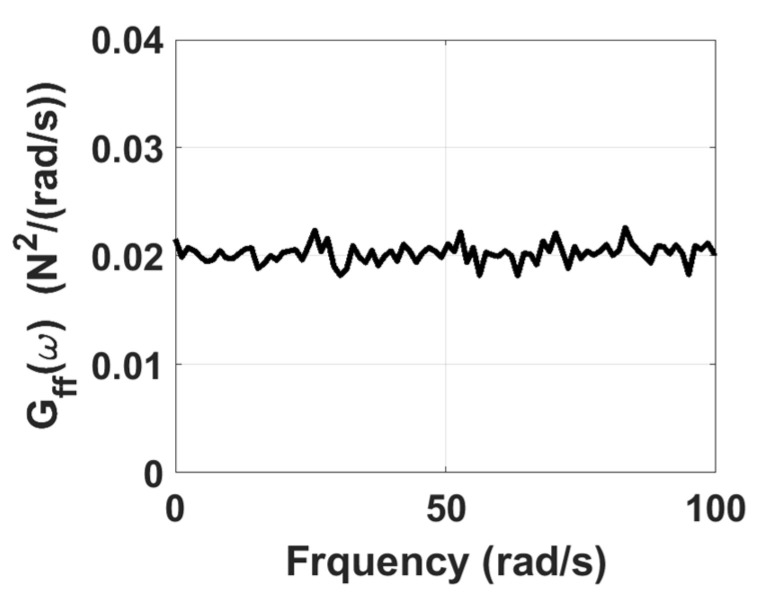
Power spectrum associated with the simulated stationary white noise served as the excitation input acting on the 6-DOF chain model.

The damping matrix $\left[C\right]$ of the system is proportional damping, i.e., the linear combination of mass matrix $\left[M\right]$ and stiffness matrix $\left[K\right]$. The stationary white noise is generated using the spectrum approximation method as a zero-mean bandpass noise, as shown in Figure 2, for which the power spectral density constant is ${0.02\;N}^{2}\cdot s/\mathrm{rad}$, and the phase angle is a random variable uniformly distributed in 0~2π. To obtain the displacement response in time domain, the sampling interval is chosen as 0.01 s, and the sampling period is 131,072 s. To simulate the situation that the system is subjected to base excitation, the system is assumed to be initially at rest, the stationary white signal excitation is applied to the sixth degree of freedom of the system, and the corresponding displacement responses of the system can be obtained using Newmark’s method.

**Comparison of computation efficiency of singular spectrum analysis**

**Figure 3:**
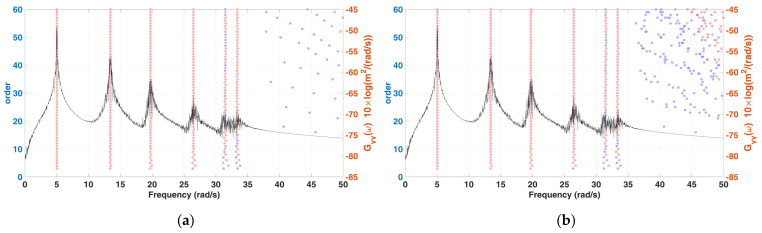
A stabilization diagram of 6-DOF chain model subjected to stationary white noise excitation (*K* = 50,000) (**a**) original SSA (**b**) modified SSA (MSSA).

To effectively obtain modal estimation results from the measured complete system signal, especially for damping identification, a longer acquisition time sample is often selected, so the corresponding singular value decomposition operation time is increased. The characteristics of the left and right singular matrices can be used to reduce the dimension of singular value solution, so as to improve the computational efficiency. Thus, the selection length of the signal to be analyzed is less affected by the computer performance. The simulation of the 6-DOF system is performed to compare the difference in computation time to compare the difference between the original and improved algorithm procedure of SSA. The computer operating environment is as follows: the central processing unit (CPU) is an Intel^®^ Core™ i7-8086K CPU, Random access memory (RAM) is 64.0 GB and the computing software is MATLAB R2020b. Moreover, the dimension L of Hankel matrix is from 3 to 60, and K is 50,000. Two stabilization diagrams of the operation results are shown in Figure 3, from which six modes can be clearly observed, and there is only subtle differences in the identification results as shown in Table 1. The computation time of the original and improved algorithm procedure of SSA are 7108.07 s and 112.20 s, respectively. The computation time before and after the improvement in SSA is increased by about 62 times, so the overall computation efficiency is significantly improved.

**Convergence analysis with various *K* (reference signal length)**

**Figure 4:**
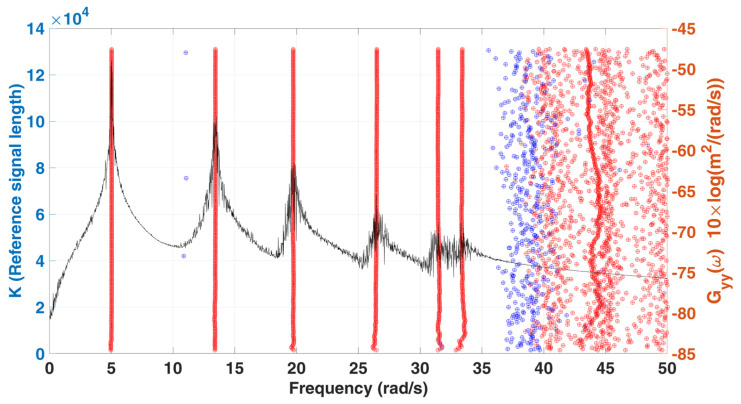
A stabilization diagram with various K of 6-DOF chain model subjected to stationary white noise excitation.

**Figure 5:**
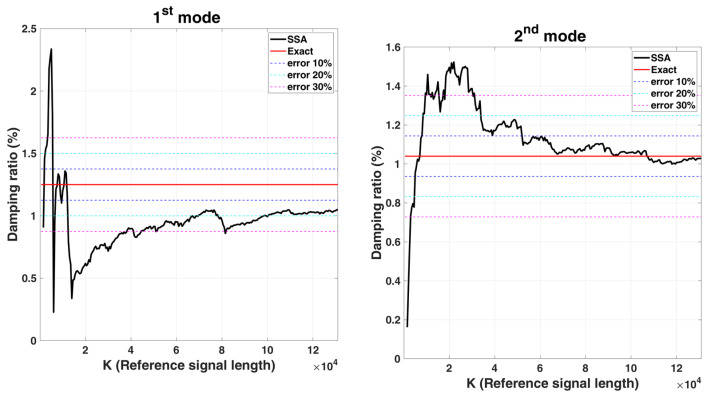

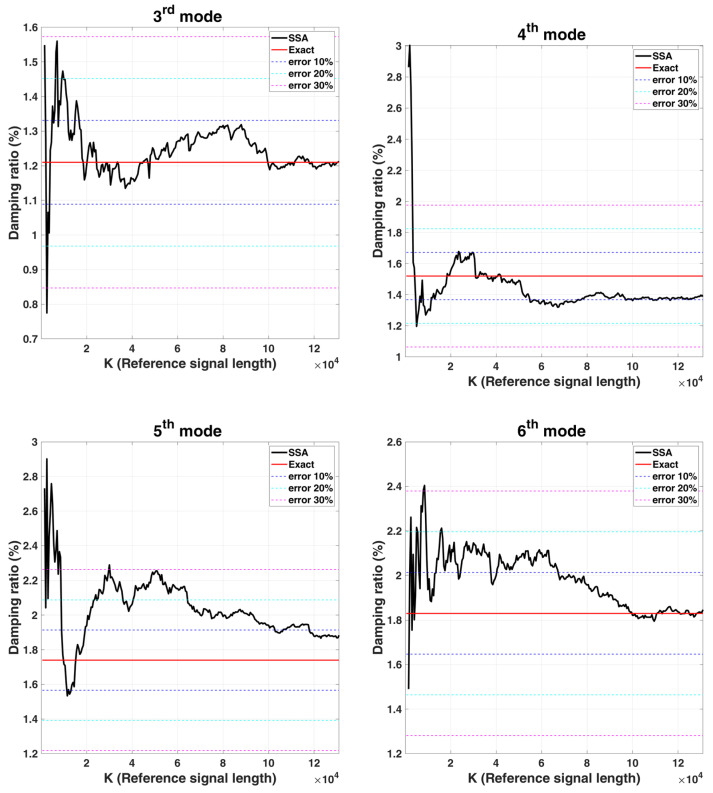
Convergence trend curves with various K for damping ratios identification results of 6-DOF chain model subjected to stationary white noise excitation.

Convergence analysis is carried out for the parameters K of the Hankel matrix. Here, the parameter L is fixed as 60, and each increment in K is 500. The stabilization diagram of the analysis is shown in Figure 4, and the corresponding convergence trend curves with various K for damping identification is shown in Figure 5. According to the stabilization diagram, it can be found that the influence of the signal length on the frequency is not significantly different from that of the damping. From the convergence trend curves with various K for damping identification, it can be found that the damping will be greatly affected by the reference signal length. In general, the longer the signal length is, the better the identification results are.

**Convergence analysis with various *L***

**Figure 6:**
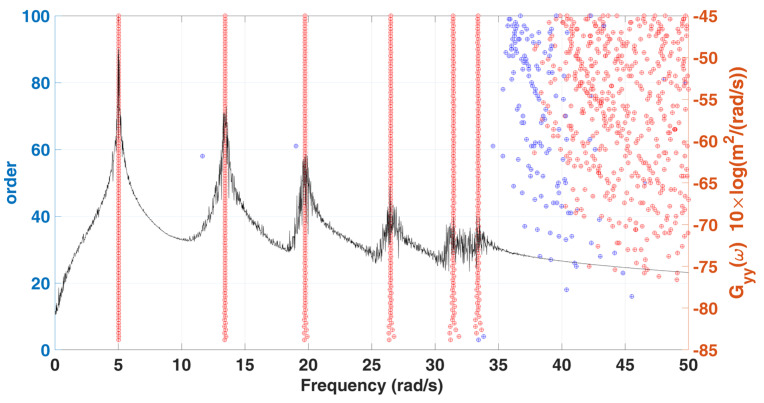
A stabilization diagram with various L of 6-DOF chain model subjected to stationary white noise excitation.

**Figure 7:**
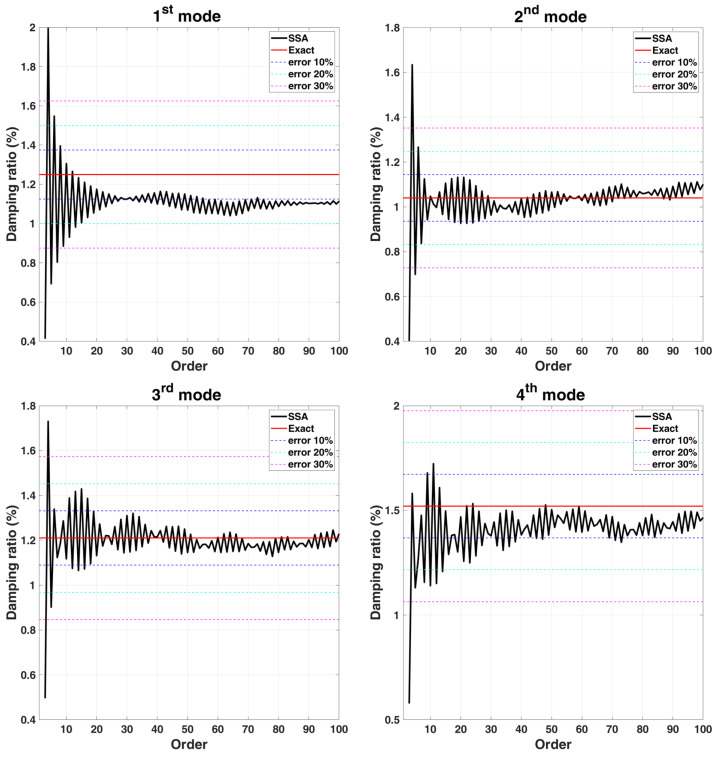

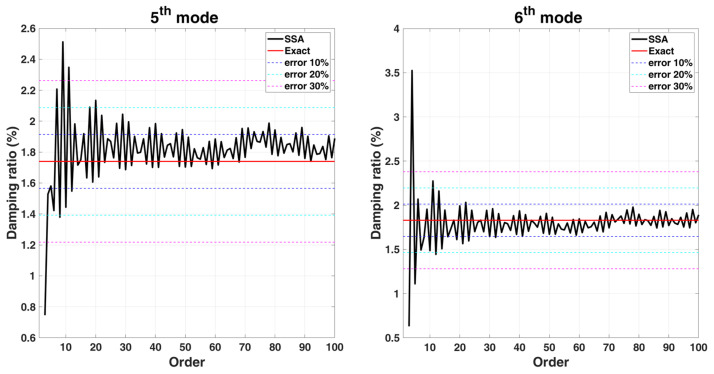
Convergence trend curves with various L for damping ratios identification results of 6-DOF chain model subjected to stationary white noise excitation.

**Figure 8:**
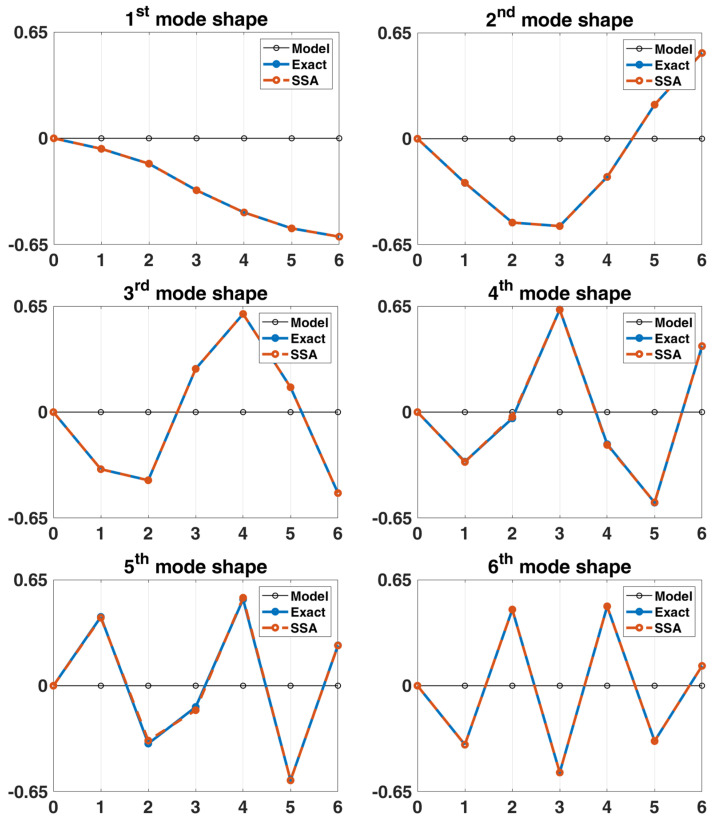
Comparison between the identified and exact mode shapes of the 6-DOF chain model system subjected to stationary white-noise input.

Convergence analysis is performed for the order L of the matrix, and its parameter K is set as m−L+1, because it can be known from the signal length convergence analysis that the larger the K is, the more stable the damping identification result is. The stabilization diagrams with various L of the 6-DOF chain model are shown in Figure 6, the corresponding damping convergence diagrams are shown in Figure 7, and the mode shape identification results are shown in Figure 8. It is observed from the stabilization diagram that the identification result of natural frequency is relatively stable, but in the damping convergence diagram, it can be observed that the damping identification error is relatively large at low orders, and the identification results have a relatively converging trend when the order is increased. Subsequent analyses use the selected parameters L=60 and K=131,013. The identification results of the 6-DOF chain model are listed in Table 2. The maximum errors in the natural frequencies and damping ratios are −0.98% and −10.45%, respectively, and the MAC values corresponding to all mode shapes are close to 1.00.

### 3.2. 2-D Truss Structure

**Figure 9:**
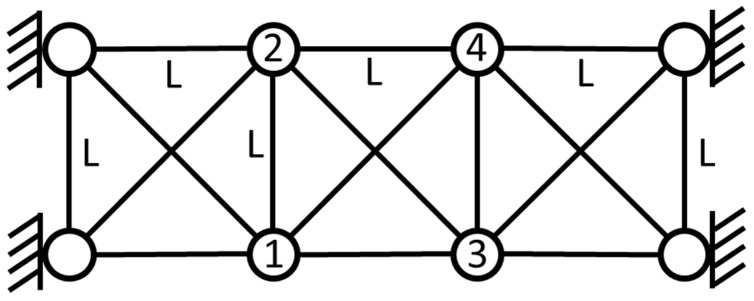
A schematic diagram of 2-D truss structure.

To examine the effectiveness of the present method for more complex structural systems, a two-dimensional truss model with proportional damping [25] is considered, as shown in Figure 9. This system has a total number of eight nodes, of which four are fully restrained, and hence the total number of active DOFs is eight (one horizontal and one vertical per each node). The mass, damping and stiffness matrices for this system are given as follows:

(30) $$\left[M\right]\;=diag\left[\begin{array}{cccccccc} 100 & 100 & 100 & 100 & 100 & 100 & 100 & 100 \end{array}\right]\mathrm{Kg}$$

(31) K =27071.1000−100000−3535.5−3535.5017071.10−1000000−3535.5−3535.50027071.10−3535.53535.5−1000000−10000017071.13535.5−3535.500−100000−3535.53535.527071.1000003535.5−3535.5017071.10−10000−3535.5−3535.5−1000000027071.10−3535.5−3535.5000−10000017071.1Nm

(32) C =0.17M + 0.001K N⋅sm

**Figure 10:**
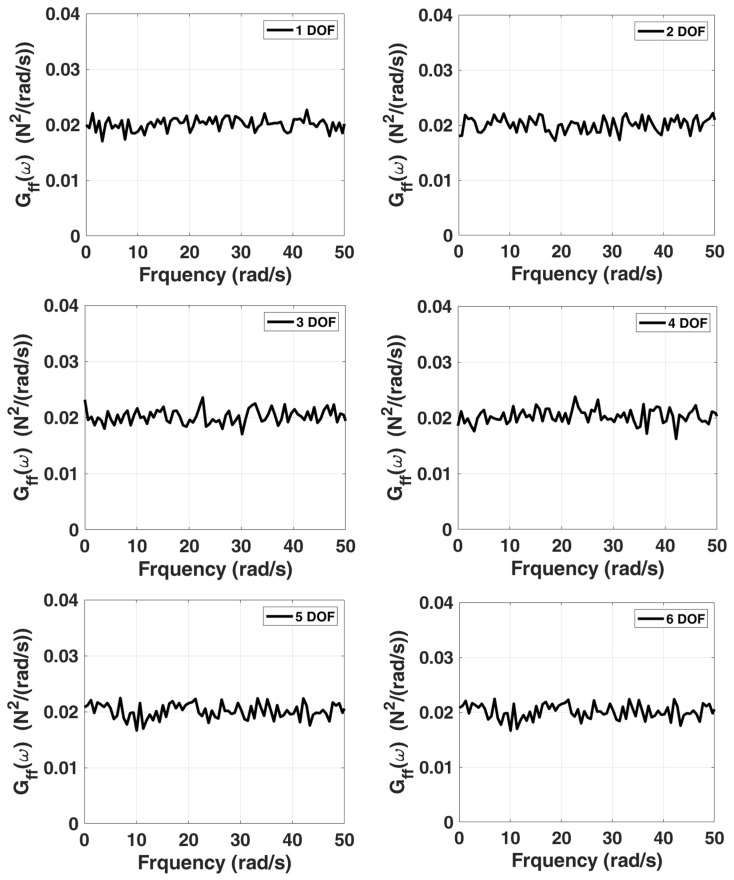

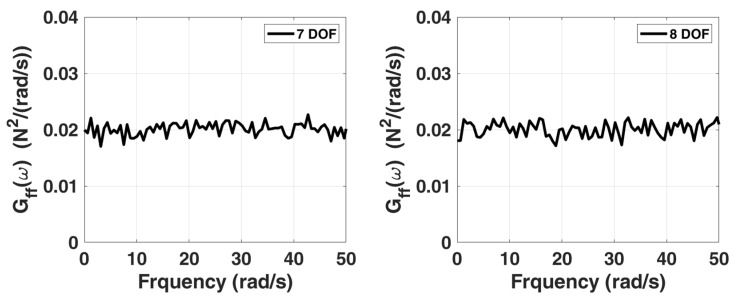
Power spectra associated with the simulated stationary white noise served as the excitation input respectively acting on the eight mass points of 2-D truss structure.

**Figure 11:**
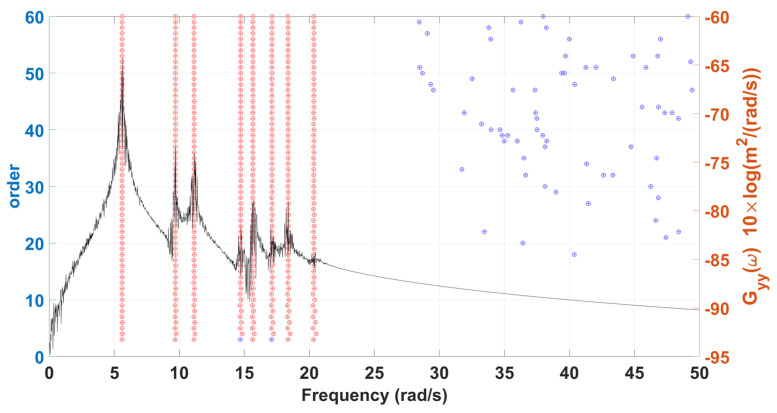
A stabilization diagram of 2-D truss structure subjected to stationary white noise excitation.

**Figure 12:**
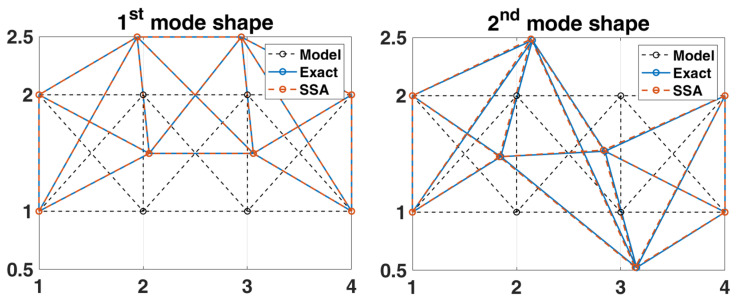

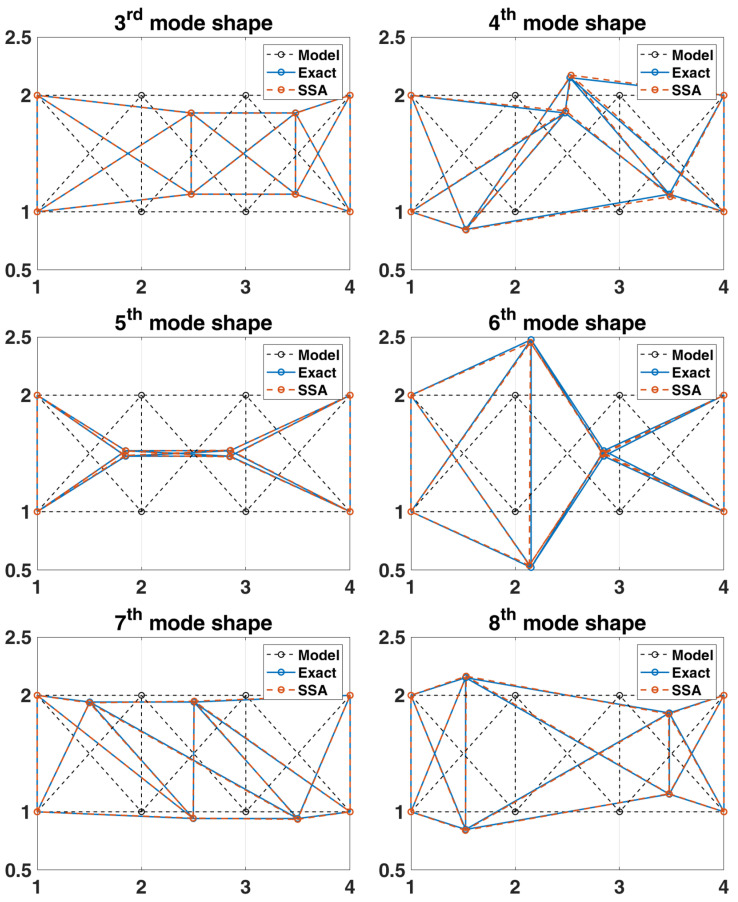
Comparison between the identified and exact mode shapes of the 2-D truss structure subjected to stationary white-noise input.

In this example, the previous stationary white-noise (with the same power spectral density constant still used, as shown in Figure 10, and a random distribution of phase angle) was used as input acting horizontally and vertically at all active DOFs of the truss model, and then the corresponding displacement responses obtained by Newmark’s method were used for modal estimation. The initial condition of this truss structural system is assumed to be at rest. The choice of the sampling interval and the sampling period are the same as the previous example. SSA is employed to analyze the simulated response data, and the corresponding stabilization diagram is shown in Figure 11. The results of modal identification with the selection of L=60 and K=131,013 are summarized in Table 3, which shows that the modal identification in this case is satisfactory. The maximum errors in natural frequencies and damping ratios are −0.43% and −16.20%, respectively. The identification results of mode shapes are shown in Figure 12. Observing the MAC values, which signify the consistency between the identified and the theoretical mode shapes, all modes are found to be identified accurately.

### 3.3. 7-DOF Model of a Sedan

**Figure 13:**
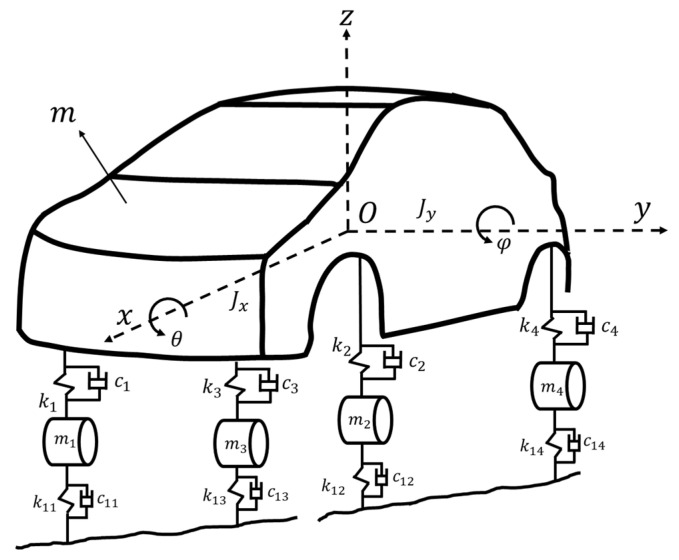
A 7-DOF model of a sedan.

To demonstrate this method effectiveness on relatively complex 3D structure systems, a sedan model with two groups of similar modes is considered [24]. The system is a 7-DOF system, as shown in Figure 13, including the bounce, pitch and roll motion of the body, as well as the bouncing motion of the four suspension systems of a sedan, in which the related parameters are shown in Ref. [24]. The mass matrix $\left[M\right]$, stiffness matrix $\left[K\right]$ and damping matrix $\left[C\right]$ of the system are expressed as follows:

(33) $$\left[M\right]=diag\left[\begin{array}{ccccccc} m & l_{y} & l_{x} & m_{1} & m_{2} & m_{3} & m_{4} \end{array}\right]$$

(34) K=[k1+k2+k3+k4−L1k1+L2k2−L1k3+L2k4−L1k1+L2k2−L1k3+L2k4L12k1+L22k2+L12k3+L22k4−L3k1−L3k2+L4k3+L4k4L1L3k1−L2L3k2−L1L4k3+L2L4k4−k1L1k1−k2−L2k2−k3L1k3−k4−L2k4−L3k1−L3k2+L4k3+L4k4−k1−k2−k3−k4L1L3k1−L2L3k2−L1L4k3+L2L4k4L1k1−L2k2L1k3−L2k4L32k1+L32k2+L42k3+L42k4L3k1L3k2−L4k3−L4k4L3k1k1+k11000L3K20k2+k1200−L4k300k3+k130−L4k4000k4+k14]Nm

(35) C =0.1M+0.001K N⋅sm

**Figure 14:**
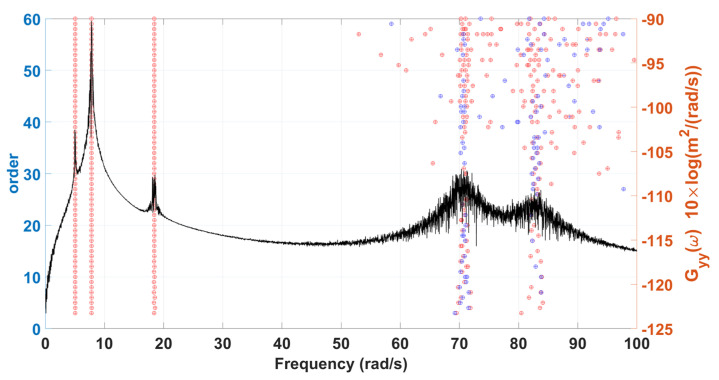
A stabilization diagram of 7-DOF model of a sedan subjected to stationary white noise excitation.

**Figure 15:**
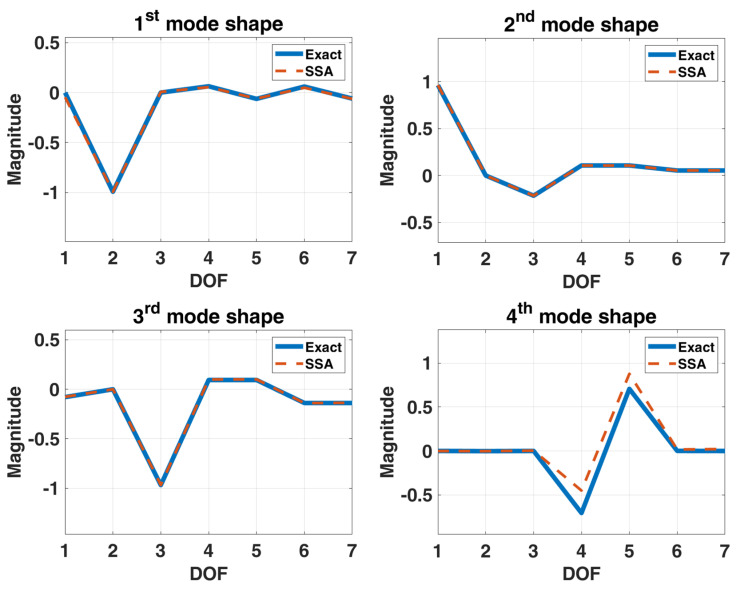

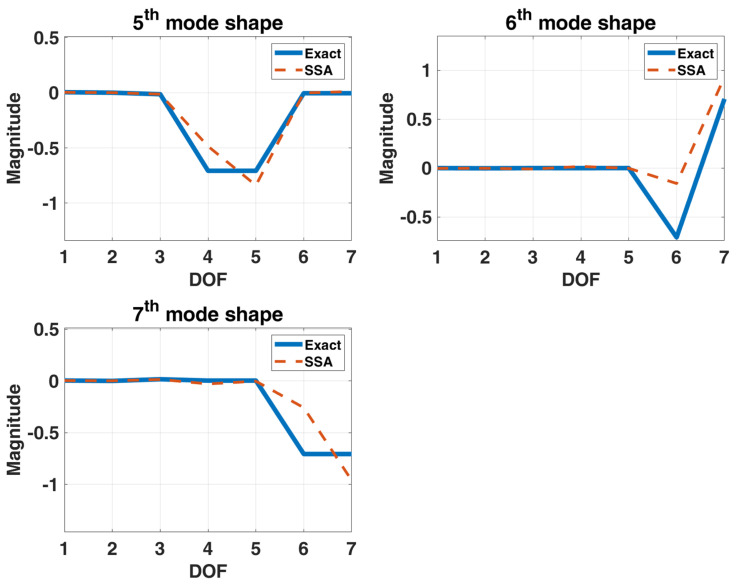
Comparison between the identified mode shapes and the exact mode shapes of the 7-DOF model of a sedan subjected to stationary white-noise input.

Considering that the vehicle will experience bumps on the road when driving, the simulated stationary white noise serves as the excitation input acts on to the DOFs of the four suspension components and bouncing of car body of a sedan. The condition of this sedan structural system is assumed to be initially at rest. The choice of the sampling interval and the sampling period are the same as the previous example. Figure 14 is the stabilization diagram of the 7-DOF sedan model, roughly showing 5 frequencies of about 5, 7, 19, 70 and 81 rad/s. Two groups of closely spaced higher modes cannot be effectively estimated from the power spectrum only. By using the distribution of poles in the stabilization diagram, it can be observed that there are two obvious poles in 70 rad/s. The order of the Hankel matrix is needed to be increased to possibly observe two severely closely spaced modes around 81 rad/s. The results of modal identification with the selection of L=60 and K=131,013 for the 7-DOF sedan model by SSA are shown in Table 4 and Figure 15. The maximum errors in natural frequencies and damping ratios are −6.89% and −26.38%, respectively, and the lowest MAC in mode shapes is 0.66.

### 3.4. 7-DOF Highly Coupled System

**Figure 16:**
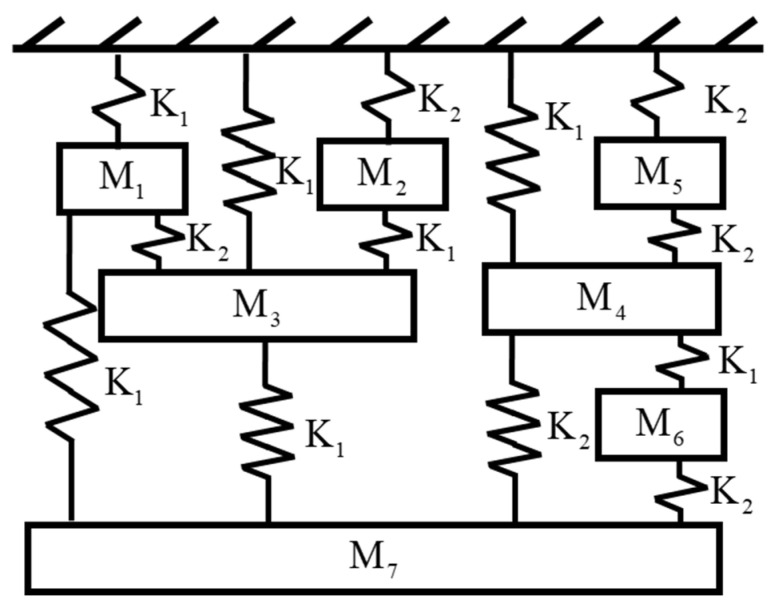
A model of 7-DOF highly coupled system.

In this case, a highly coupled system [26] is used to verify the effectiveness of the proposed method. A schematic representation of this model is shown in Figure 16. The mass matrix $\left[M\right]$, stiffness matrix $\left[K\right]$ and damping matrix $\left[C\right]$ of the system are expressed as follows:

(36) $$\left[M\right]=\left[\begin{array}{ccccccc} 1 & 0 & 0 & 0 & 0 & 0 & 0\\ 0 & 1 & 0 & 0 & 0 & 0 & 0\\ 0 & 0 & 1 & 0 & 0 & 0 & 0\\ 0 & 0 & 0 & 1 & 0 & 0 & 0\\ 0 & 0 & 0 & 0 & 1 & 0 & 0\\ 0 & 0 & 0 & 0 & 0 & 1 & 0\\ 0 & 0 & 0 & 0 & 0 & 0 & 1 \end{array}\right]Kg$$

(37) K=400−20000−10030−100000−20−1050000−1000060−20−10−20000−204000000−10030−20−100−10−200−2060×103Nm

(38) $$\left[C\right]\;=0.2\left[M\right]\;+\;0.0003\left[K\right]$$

**Figure 17:**
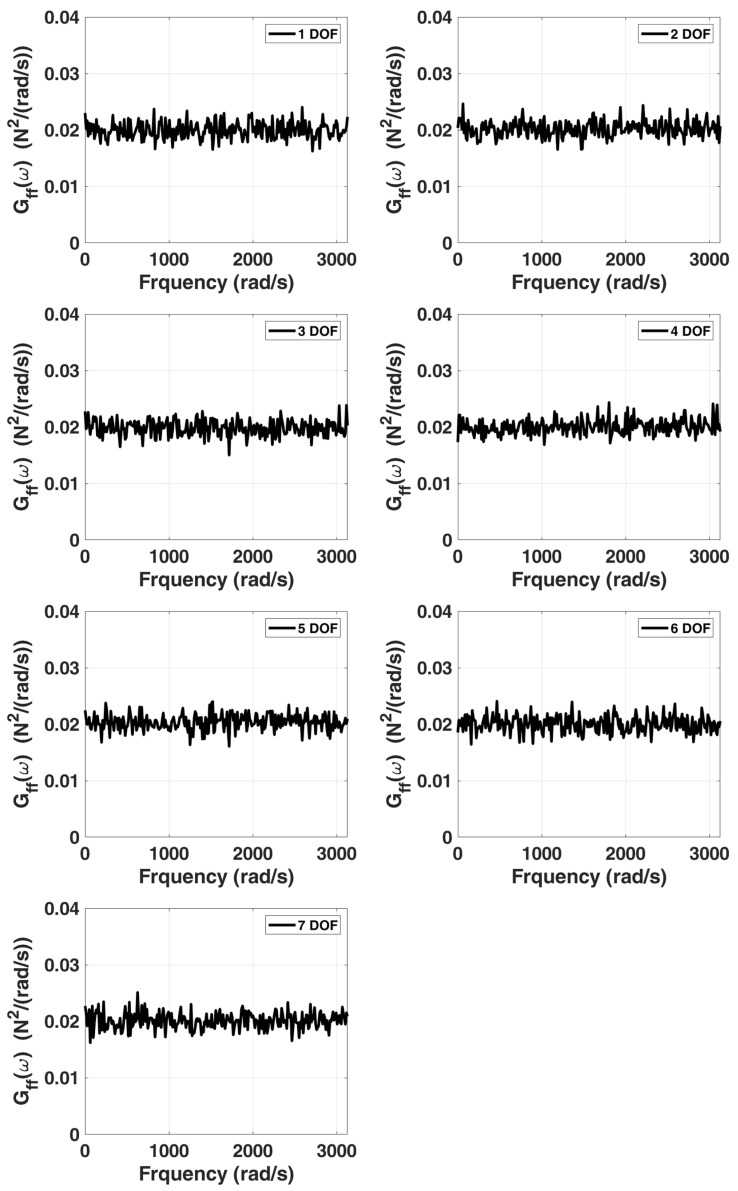
Power spectra associated with the simulated stationary white noise served as the excitation input respectively acting on each mass point of a 7-DOF highly coupled system.

**Figure 18:**
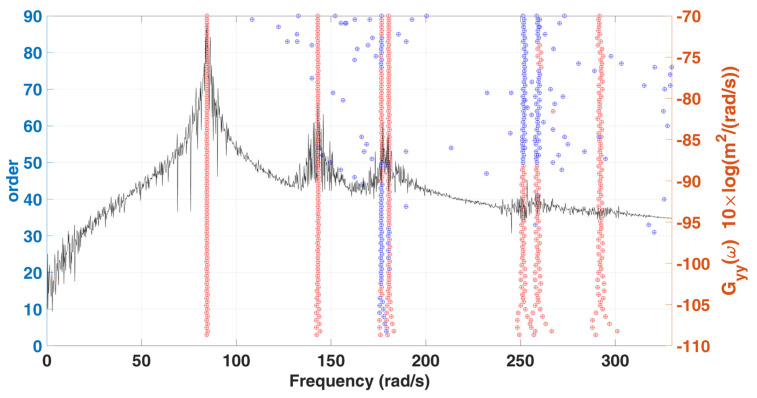
A stabilization diagram of 7-DOF highly coupled system subjected to stationary white noise excitation.

**Figure 19:**
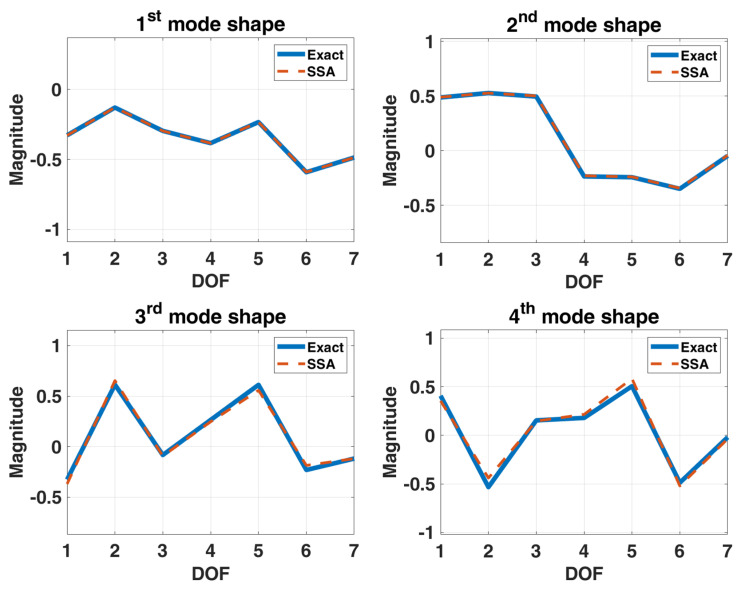

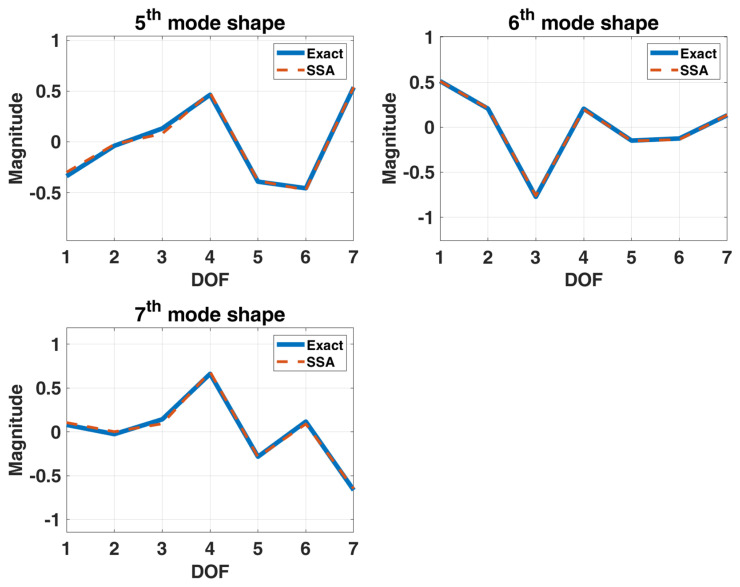
A model of 7-DOF highly coupled system.

In this example, we still use the previous stationary white-noise (with the same power spectral density constant as shown in Figure 17 and random distribution of phase angle) as input acting all DOFs of this model, and then the corresponding displacement responses obtained by Newmark’s method are used for modal estimation. We assume that the initial condition of this truss structural system is at rest. The choice of the sampling interval is chosen as 0.001 s, and the sampling period is 131.072 s. SSA is employed to analyze the simulated response data, and the corresponding stabilization diagram is shown in Figure 18. Table 5 and Figure 19 summarize the results of the modal identification, with the selection of L=90 and K=130983 in this case, and present the well-identified modal parameters of the system. The maximum errors in natural frequencies and damping ratios are −0.98% and 28.90%, respectively, and the lowest MAC in mode shapes is 0.98.

## 4. Conclusions

The singular spectrum analysis (SSA) in the present paper can be effectively applicable to modal estimation from stationary response only. In this paper, the system response data is composed of Hankel matrix, and the appropriate factorization of data matrix under consideration is to process through embedding, singular value decomposition (SVD), grouping, and averaging in SSA. The dynamic information of systems is effectively extracted, the influence of external force and noise on the data matrix is reduced and the corresponding observability matrix of the system to be identified can be obtained. Then, the modal parameters are estimated through the appropriate factorization of the observability matrix. In addition, the characteristics of the left and right singular matrices in SVD can be employed to effectively reduce the dimension of the data matrix. Therefore, the computation efficiency of the response data matrix constructed from long-time data samples is significantly improved and without the restriction of computer performance to affect the length of the chosen response data. In additional, when SSA is applicable to identify modal parameters, increasing the system order can make SVD more effective in removing non-structural information, but it may yield some fictitious modes due to numerical computation; the stabilization diagram is employed to determine whether they are fictitious modes and to determine the system modes.

## Figures and Tables

**Table 1:** Identification results of natural frequency and damping ratio of 6-DOF chain model through the original and modified SSA.

Natural Frequency (rad/s)						
Mode	Exact	SSA	Error (%)	MSSA	Error (%)	SSA vs MSSA Diff. (%)
1	5.03	5.04	0.20	5.04	0.20	$1.88\;\times {\;10}^{-8}$
2	13.45	13.43	−0.15	13.43	−0.15	$1.69\;\times {\;10}^{-7}$
3	19.8	19.71	−0.45	19.71	−0.45	$-1.47\;\times {\;10}^{-7}$
4	26.69	26.46	−0.86	26.46	−0.86	$-9.68\;\times {\;10}^{-8}$
5	31.66	31.49	−0.54	31.49	−0.54	$1.96\;\times {\;10}^{-7}$
6	33.73	33.36	−1.10	33.36	−1.10	$1.12\;\times {\;10}^{-7}$
**Damping ratio (%)**						
**Mode**	**Exact**	**SSA**	**Error (%)**	**MSSA**	**Error (%)**	**SSA vs MSSA** **Diff. (%)**
1	1.25	0.91	−27.20	0.91	−27.20	$-1.40\;\times {\;10}^{-5}$
2	1.04	1.18	13.46	1.18	13.46	$-6.30\;\times {\;10}^{-6}$
3	1.24	1.23	−0.81	1.23	−0.81	$1.24\;\times {\;10}^{-5}$
4	1.52	1.49	−1.97	1.49	−1.97	$-1.80\;\times {\;10}^{-5}$
5	1.74	2.24	28.74	2.24	28.74	$2.07\;\times {\;10}^{-5}$
6	1.83	2.08	13.66	2.08	13.66	$2.26\;\times {\;10}^{-5}$

**Table 2:** Modal estimation results of 6-DOF chain model.

	Natural Frequency (rad/s)			Damping Ratio (%)			
Mode	Exact	SSA	Error (%)	Exact	SSA	Error (%)	MAC
1	5.03	5.03	−0.02	1.25	1.11	−10.45	1.00
2	13.45	13.44	−0.10	1.04	1.10	5.43	1.00
3	19.80	19.74	−0.31	1.24	1.23	−1.07	1.00
4	26.69	26.51	−0.66	1.52	1.47	−3.72	1.00
5	31.66	31.43	−0.73	1.74	1.89	8.50	1.00
6	33.73	33.39	−0.98	1.83	1.89	3.08	1.00

**Table 3:** Modal estimation results of 2-D truss model.

	Natural Frequency (rad/s)			Damping Ratio (%)			
Mode	Exact	SSA	Error (%)	Exact	SSA	Error (%)	MAC
1	5.59	5.60	0.26	1.80	1.59	−11.94	1.00
2	9.74	9.71	−0.33	1.36	1.33	−2.42	1.00
3	11.14	11.14	−0.03	1.32	1.48	12.10	1.00
4	14.74	14.72	−0.11	1.31	1.10	−16.20	1.00
5	15.70	15.66	−0.24	1.33	1.19	−10.62	1.00
6	17.17	17.12	−0.29	1.35	1.19	−12.19	1.00
7	18.42	18.35	−0.37	1.38	1.33	−3.94	1.00
8	20.43	20.34	−0.43	1.44	1.42	−1.51	1.00

**Table 4:** Modal estimation results of 7-DOF model of a sedan.

	Natural Frequency (rad/s)			Damping Ratio (%)			
Mode	Exact	SSA	Error (%)	Exact	SSA	Error (%)	MAC
1	5.03	5.05	0.45	1.25	0.92	−26.35	1.00
2	7.82	7.81	−0.08	1.03	1.03	−0.33	1.00
3	18.47	18.40	−0.36	1.19	1.24	4.41	1.00
4	73.79	70.25	−4.79	3.76	2.77	−26.38	0.91
5	73.87	71.02	−3.86	3.76	3.42	−9.05	0.93
6	87.83	81.79	−6.87	4.45	3.82	−14.15	0.66
7	88.07	82.00	−6.89	4.46	3.94	−11.59	0.75

**Table 5:** Modal estimation results of a 7-DOF highly coupled system.

	Natural Frequency (rad/s)			Damping Ratio (%)			
Mode	Exact	SSA	Error (%)	Exact	SSA	Error (%)	MAC
1	84.11	84.55	0.53	1.38	1.78	28.90	1.00
2	143.56	143.05	−0.36	2.22	2.40	7.99	1.00
3	177.02	176.66	−0.20	2.71	2.38	−12.06	0.99
4	181.38	180.39	−0.55	2.78	2.52	−9.50	0.98
5	252.24	251.34	−0.36	3.82	3.68	−3.57	0.99
6	260.03	258.59	−0.55	3.94	4.35	10.35	0.98
7	294.68	291.79	−0.98	4.45	3.99	−10.36	0.99

## Data Availability

Not applicable.
